# Regulations in the Indiana Medical College

**Published:** 1847-10

**Authors:** 


*Regulations in the Indiana Medical College.*—By the by-laws of this institution, candidates after having passed a satisfactory examination, are required to publicly assent to the following promise before the degree is conferred: "You do solemnly promise that you will, to the utmost of your abilities, exert your influence for promoting the welfare and respectability of the profession: That you will demean yourselves honorably in the practice thereof: That you will not put forth any nostrum or secret method of cure, and that you will not publish any matter or thing derogatory of the profession."—*Western Lancet.*

